# Accuracy of genome-enabled polygenic risk score prediction of cruciate ligament rupture risk in the Labrador Retriever

**DOI:** 10.3389/fvets.2025.1625953

**Published:** 2025-08-26

**Authors:** Benjamin Miranda, Mehdi Momen, Susannah J. Sample, Peter Muir

**Affiliations:** Comparative Orthopaedic & Genetics Research Laboratory, Department of Surgical Science, School of Veterinary Medicine, University of Wisconsin-Madison, Madison, WI, United States

**Keywords:** cruciate ligament rupture, dog, genome-wide association study, genomic prediction, polygenic risk score prediction, Labrador Retriever

## Abstract

**Introduction:**

Canine cruciate ligament rupture (CR) is a common, complex, polygenic, orthopaedic disease in dogs that results in serious financial burden and patient morbidity even in the face of surgical correction. The goal of this study was to evaluate the clinical utility of CR polygenic risk score (PRS) prediction models using genome-wide SNP data from a large reference population of Labrador Retriever dogs.

**Methods:**

Using 10-fold cross-validation and an independent validation population, we assessed Bayesian and machine learning models with and without covariates using both genome-wide SNPs as well as genic SNPs. Models were tuned by optimizing numbers of CR risk SNPs selected by genome-wide association and adjusting posterior probability thresholds to maximize prediction accuracy.

**Results:**

Models that included clinical covariates (sex, neuter status, age, weight, withers height, as well as the first 10 principal components from the genetic relationship matrix) universally yielded higher accuracy up to 88.5% compared to 77% without covariates. Prediction accuracy for some models was reduced when only genic SNPs were used suggesting SNPs in non-coding regions could influence the CR disease risk.

**Discussion:**

Our results confirm that PRS models provide sufficient predictive accuracy for clinical application in veterinary medicine and offer a viable, early-life screening tool for personalized care and selective breeding to reduce CR incidence in high-risk breeds. Our results further confirm that CR is a complex polygenic disease in which genome-wide risk SNPs influence disease pathogenesis.

## Introduction

Canine cruciate ligament rupture (CR) is one of the most common orthopaedic diseases encountered in veterinary medicine (1). The disease often results in serious long-term sequelae such as reduced mobility from osteoarthritis even with surgical stabilization of the stifle since osteoarthritis is typically established at diagnosis (2). With a high rate of contralateral rupture, CR results in a high patient morbidity and a high economic burden to owners (3, 4). CR is a complex polygenic disease in which both environmental and genetic risk contribute to disease progression (5). Some of these factors include breed predisposition (6), ligament matrix degeneration (7), obesity (8), conformation (8), and joint immune responses (7). In addition, ligament rupture is usually a consequence of complex pathogenesis where polygenic effects on various physiological pathways affect cruciate ligament homeostasis in different ways that promote fatigue injury to collagen fibers with progressive fiber rupture in the presence of synovitis as the cause of the majority of non-contact CR rather than a single cycle mechanical overload of the cranial cruciate ligament (5). The concept that CR is a heritable disease rather than an injury aligns with a growing body of evidence in the human literature regarding non-contact ACL rupture (5, 9, 10).

The prevalence of CR is breed dependent with heritability estimates ranging from 0.27–0.85 in dogs (11–14). Breeds with high prevalence, such as the Labrador Retriever, Rottweiler, and Newfoundland, have a concentration of risk loci because of breed selection (15, 16). Genomic studies in dogs have shown CR is highly polygenic in the Labrador Retriever (5, 11). Genome-wide association studies have identified few large effect and numerous small effect genetic variants suggesting CR is primarily a polygenic disease (5, 17). Current heritability estimates in the Labrador Retriever (0.52–0.63) suggest CR is a disease with moderate to high heritability (5). Studies of the genetic architecture of CR in the Labrador Retriever have also shown that risk of CR is influenced by coat color (18). Many risk genes are also shared with human ACL rupture (5).

For complex heritable diseases, polygenic risk score (PRS) prediction enables quantification of an individual's risk by assuming all single nucleotide polymorphisms (SNPs) are disease-associated risk variants even if their effect is very small (11, 19). These variants in combination influence disease risk and can be analyzed by risk models to estimate the probability of an individual developing the disease over their lifetime (20). So, a PRS value represents the heritable risk of developing a disease in an individual based on the total number of significant genetic variants they have (21). PRS prediction is widely used in the study of human complex polygenic disease and is now being increasingly studied in companion animals in veterinary medicine (5, 21).

In the current study, our goal was to validate PRS prediction of the risk of CR in the Labrador Retriever, as the Labrador is one of the high-risk breeds with an increased prevalence above the general population at 5.79% (6). Our previous research has generated a large reference population of Labrador Retrievers accurately phenotyped as CR cases or controls, enabling definitive estimates of heritability, genetic architecture, and initial PRS prediction using cross validation in this reference population (5). The purpose of the present study was to continue clinical development of PRS prediction of risk of CR in the Labrador Retriever by using a new validation population to confirm PRS prediction has sufficient accuracy for clinical use (5, 20).

## Materials and methods

### Data collection and phenotyping

Client-owned Labrador Retriever dogs were recruited at the University of Wisconsin-Madison School of Veterinary Medicine through online advertising, local, and national breed clubs for the validation group. All procedures were performed in accordance with the recommendations in the Guide for the Care and Use of Laboratory Animals of the National Institutes of Health and the American Veterinary Medical Association and IACUC approval (V5463). All owners gave informed consent. Purebred status was confirmed from a pedigree for each dog. Relatedness between individuals was screened via pedigree review and siblings were excluded to reduce Type 1 error rates. Dogs were phenotyped by orthopaedic exam and lateromedial stifle radiographs. Dogs were considered a case if they had CR diagnosed by a veterinarian with most cases having their CR confirmed during surgical stifle stabilization. Labrador Retriever dogs were classified as a control if >8 years of age, had both stifles palpated as stable by a veterinarian, and no evidence of stifle effusion or osteophytosis on stifle radiograph that would be indicative of a CR (22). This age threshold was chosen because Labrador Retrievers ≥8 years have an ~6% chance of experiencing CR (23). Age, weight, withers height, sex, neuter status, and coat color were also recorded. If a control dog subsequently developed a CR, the phenotype was updated.

### Sample populations and SNP genotyping quality control

DNA was obtained from blood or saliva samples. SNP genotyping was performed using Illumina CanineHD BeadChip containing ~230,000 SNPs across the canine genome (CanFam3.1). The reference or training population (TRN) group of Labrador Retriever dogs contained 1,006 dogs (440 cases, 556 controls). The meta dataset was made up of dogs recruited at UW-Madison 719 Labrador Retrievers (326 cases, 383 controls) and the second was provided by Cornell University 287 Labrador Retrievers (114 cases, 173 controls) (5). The covariate phenotypes were not available for the dataset from Cornell University. The validation or testing group (TST) of Labrador Retrievers consisted of 52 dogs (24 cases, 28 controls). Within cases in the TST group, there were 8 neutered males, 3 intact males, 12 ovariohysterectomized females, and 1 intact female. Within controls, there were 7 neutered males, 7 intact males, 13 ovariohysterectomized females, and 1 intact female.

Quality control filtering of genotypic data was performed using PLINK v1.9 software (24). Samples with a genotyping call rate below 95% were excluded. SNPs were removed from the dataset if they had a minor allele frequency (MAF) < 0.01, had a genotyping call rate ≤ 95%, or if they deviated from Hardy-Weinberg proportions at a *P* < 1E-06. Missing genotypes were imputed using Beagle 5.4 software (25, 26). SNP data quality control resulted in 142,071 SNPs remaining.

### Experimental design for polygenic risk score prediction

**Figure 1:**
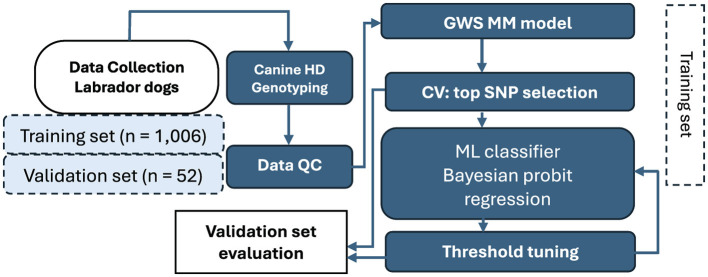
Flowchart illustrating the workflow for polygenic risk score prediction and threshold tuning of case-controls for cruciate ligament rupture in the Labrador Retriever. Data from 1,064 dogs were used as the reference training set (*n* = 1,006) and SNP effects were tested on an independent validation set (*n* = 52). All samples were genotyped by Canine HD BeadChip and quality controlled. Genome-wide selection (GWS) using a mixed-model (MM) approach was applied, followed by 10-fold cross-validation (CV) for top risk SNP selection. Predictive modeling employed machine learning classifiers, Bayesian probit regression models, and ensemble logistic regression, with threshold tuning finalized using the validation dataset. Ten-fold cross validation was then rerun after optimization of risk SNP selection and threshold tuning.

The bioinformatics approach for our analysis is summarized in Figure 1. The TRN group of 1,006 Labrador Retriever dogs was used for model fitting. The TST group of 52 Labrador Retriever dogs was used as an independent validation sample. Each dog in the TST group had a predicted phenotype from their PRS value and a true CR case and control phenotype. Eight statistical models composed of four Bayesian regression models and four machine-learning models were used to estimate the predicted phenotype. The Bayesian models were Bayesian Ridge Regression (BRR), Bayesian Lasso (BL), Bayes B (BB), and Bayes C (BC) (5, 20). All the Bayesian models were fitted using the BGLR package (27). The four machine-learning models were Least Absolute Shrinkage and Selection Operator (LASSO), Support Vector Machine (SVM), Random Forest (RF), and Elastic Net (EN) (28). EN and LASSO were fitted using the glmnet function from the glmnet R-package (29). RF was implemented using the R-package “wsrf” (30) and SVM used the e1071 package (31).

### Ten-fold cross validation

Initially, 10-fold cross validation was performed using the reference TRN group. The data were randomly partitioned into 10-folds, with nine of the folds used for model training and the 10^th^ used as a test set. Each test set was assessed in turn until all 10-folds had been evaluated. The partition scheme used was like that in Baker et al. (20). The advantage of multiple-fold cross validation is that it allows the training dataset to remain large without sacrificing a portion of the dataset for testing. The predictions were aggregated from the 10 folds and averaged across the runs.

Prediction performance for each model was assessed using accuracy (ACC) and the area under the receiver operator characteristic (ROC) curve (AUC). After obtaining a posterior probability for all folds, we computed the prediction accuracy metrics. Clinical covariates were included in our cross-validation analysis and were sex, neuter status, weight, age, and withers height, as well as 10 principal components (PCs) from the genetic relationship matrix using all dogs. We also computed a genomic relationship matrix (GRM) and verified that the average genomic relationship between the reference (TRN) and validation (TST) populations was close to zero (Mean = −0.01, SD = 0.038). ACC values were also calculated using the tuned posterior probability thresholds (see below).

### Optimization of risk SNP selection for each statistical model

Because each statistical model has a different analytical approach, different models often perform optimally with differing numbers of CR risk SNPs. Risk SNPs were selected based on strength of association with CR by genome-wide association study (GWAS) using a threshold mixed model for a binary trait (32). Cross-validation models were run with different numbers of risk SNPs to determine the optimum performance.

### Optimization of the posterior probability threshold for distinguishing cases and controls

After obtaining the posterior probability for each individual dog in the validation TST group using the eight models, we determined the optimum threshold that maximized CR risk prediction accuracy and best distinguish cases from control. Youden's J statistic (P^*^), ACC, and geometric mean (gMean) were used as metrics for threshold optimization (33). The Youden's J statistic computed as P^*^ = |FPR + TPR – 1| where FPR is the false positive rate and TPR is the true positive rate. FPR represents the proportion of incorrectly identified positive results. FPR = FP/(FP + TN) where FP (False Positive) are the positive results incorrectly predicted, and TN (True Negative) are the negative results correctly predicted. TPR represents the proportion of correctly identified positive results and is also known as Sensitivity. TPR = TP/(TP + FN) where TP (True Positive) are the positive results correctly predicted, and FN (False Negative) are the negative results incorrectly predicted. The gMean, or geometric mean, a metric that is particularly valuable in binary classification tasks, focuses on the balance between the TPR (Sensitivity) and the TNR (Specificity); gMean = $\sqrt{\left(TPR^{*}TNR\right)}$ (34). TNR is the true negative rate, also known as Specificity, and represents the proportion of correctly identified negative results. Furthermore, the predictive accuracy of models (ACC) defined as $ACC=\frac{\left(TP+TN\right)}{\left(TP\;+\;TN\;+\;FP\;+\;FN\right)}$, was evaluated for all thresholds. Then, a grid search was performed, looking at a range of posterior probabilities from 0 to 1 with 0.025 intervals to find the most reliable cut off threshold for distinguishing CR cases from controls according to the posterior probability.

### Genic ontology and CR prediction according to genic region

A gene list was collected by referencing all genes related to CR from previous publications as summarized in Table 3 in Baker et al. (17). A pathway and gene ontology (GO) analysis was then performed using the most significant GO terms or wiki pathways associated with CR to verify each gene's relationship with the biology of CR. The GO is a database compiling the biological background of genes and gene products across species. Next, all genetic variants located within the gene list's genic regions without flanking regions were extracted for prediction purposes. Genic regions were identified for each gene using the UCSC Genome Browser with the transcription and coding start and stop coordinates, respectively, used to define each gene location. The TRN group of 1,006 Labrador Retriever dogs was used for model training and the TST group of Labrador Retriever dogs for validation. The eight previously used models were used to measure all predictive performance metrics.

### Assessment of CR prediction accuracy

A posterior probability from each model was generated for each dog in the TST group. Ensembles of the Bayesian models or machine learning models were also used to generate an average posterior probability for individual dog risk prediction as a CR case or control. This predicted phenotype was then compared to the true phenotype of each dog. AUC and three different coefficients of determination (R^2^) metrics were calculated to assess the predictive performance of different model scenarios. AUC was calculated using the pROC R package (34–38). The three R^2^ values were Cox and Snell's R^2^ (${\text{R}}_{C\&S}^{2}$) (39–41), Efron's R^2^ (${\text{R}}_{ef}^{2}$) (42), and Nagelkerke's R^2^ (${\text{R}}_{nag}^{2}$) (43). We evaluated all models with and without considering covariates and considered either whole genome SNPs or genic only SNPs. The covariate variables we considered were sex, neuter status, weight, age, and withers height as well as 10 principal components from the genetic relationship matrix. The TRN group of Labrador Retrievers were used as the training set and the TST group of dogs for validation.

## Results

### Optimization of CR risk SNPs for PRS prediction

The top GWAS SNPs were ranked based on *P*-value, and different percentages of top SNPs were selected to further evaluate model performance based on analysis using 10-fold cross-validation. The optimal predictive ability for CR risk varied across statistical models. The best predictive performance with all Bayesian models was obtained using 30% of the SNPs (42,621 SNPs). The best performance with RF was achieved using the top 1% of the SNPs (1,420 SNPs), while the best predictive performance for EN and LASSO was obtained using 2% of the SNPs (2,841 SNPs). The best performance with SVM was obtained using 7% of the SNPs (9,944 SNPs).

### Model performance using top GWAS SNPs, threshold optimization with the test group, and subsequent 10-fold cross validation in the reference group

**Figure 2:**
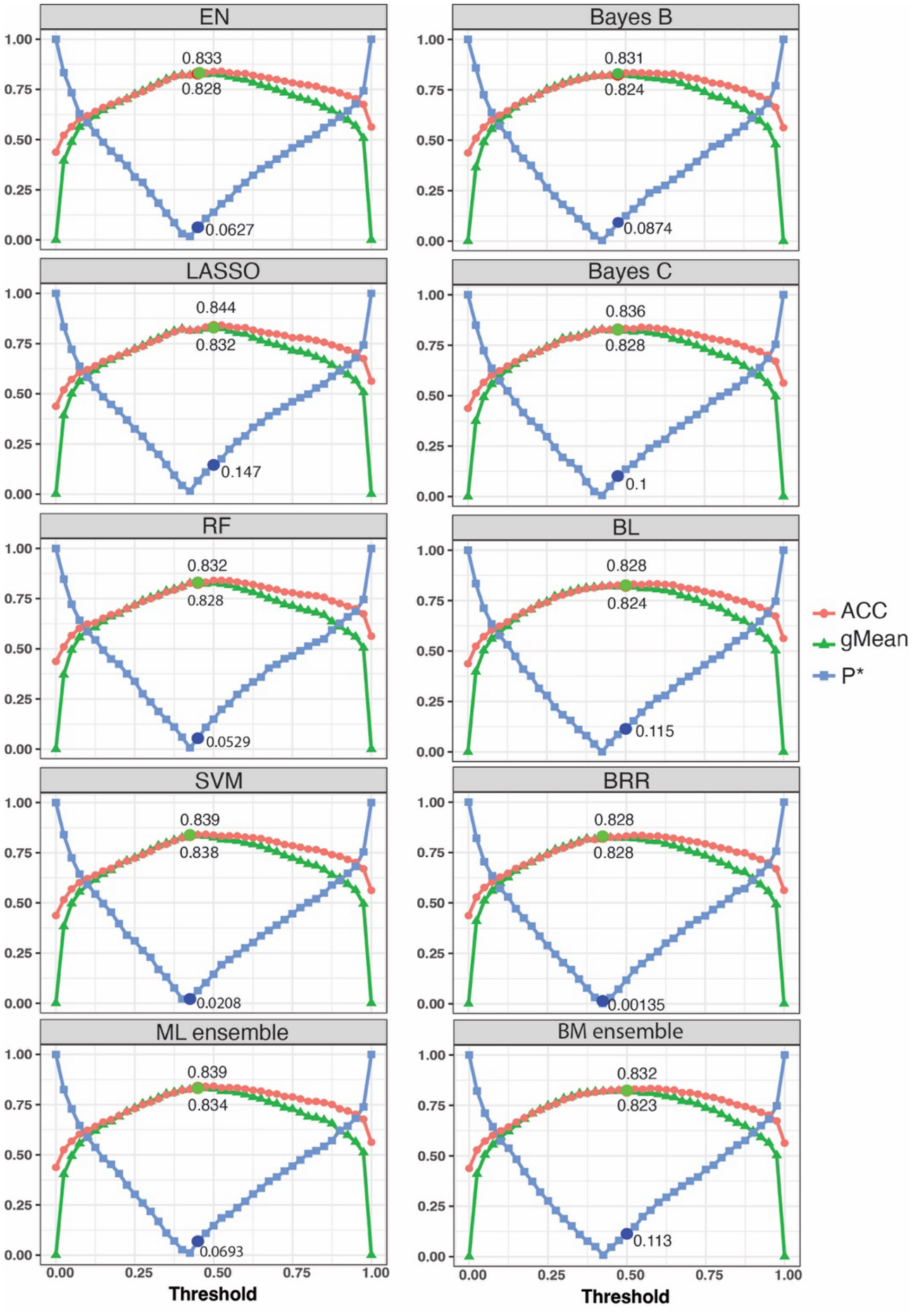
A grid search was performed to identify the optimum threshold range and evaluate model performance for prediction of cruciate ligament rupture case status. Each data point represents a threshold ranging from 0 to 1 with 0.025 intervals. Lower P* values aligned with higher ACC and gMean values. The circle dots show the optimum point for each model. EN, Elastic Net; LASSO, Least Absolute Shrinkage and Selection Operator; RF, Random Forest; SVM, Support Vector Machine; ML, machine learning; BL, Bayesian Lasso; BRR, Bayesian Ridge Regression. The analysis used a training group of 1,006 Labrador Retrievers for model training and a test group of 52 Labradors for prediction optimization.

We used the P^*^, ACC, and gMean metrics for model tuning to determine the optimum threshold points for each model using Labrador Retriever dogs in the TST group. We considered the threshold as optimal when the ACC and gMean were highest and P^*^ was minimum. Optimal thresholds ranged from 0.425 to 0.5 (Figure 2, Table 1). Among machine learning models, the highest ACC was achieved by the LASSO machine learning model after model tuning (0.844) and the highest AUC was achieved with the SVM model (0.838) at P^*^ = 0.021 (Table 1). For Bayesian models, the highest ACC was observed with Bayes C (0.836) at P^*^ = 0.1 (Table 1), which also achieved the highest AUC (0.83).

**Table 1:** Accuracy of Bayesian and machine learning statistical models for prediction of cruciate ligament rupture risk using polygenic risk scores in the Labrador Retriever reference population using 10-fold cross validation, top genome-wide risk SNPs, and tuned posterior probability thresholds.

**Model**	**Optimal threshold after tuning**	P^*^	**gMean**	**ACC**	**AUC**	** $R_{nag\;}^{2}$ **	** $R_{c\&s}^{2}$ **	** $R_{ef}^{2}$ **
RF	0.45	0.053	0.828	0.832	0.829	0.571	0.426	0.485
SVM	0.425	0.021	0.838	0.839	0.838	0.582	0.434	0.495
LASSO	0.5	0.147	0.832	0.844	0.835	0.579	0.432	0.491
EN	0.45	0.063	0.828	0.833	0.829	0.581	0.433	0.493
ML_Ens	0.45	0.069	0.834	0.839	0.835	0.585	0.436	0.496
BRR	0.425	0.001	0.828	0.828	0.828	0.589	0.439	0.501
BL	0.5	0.115	0.824	0.828	0.826	0.591	0.441	0.502
BayesB	0.475	0.087	0.824	0.831	0.826	0.588	0.439	0.500
BayesC	0.475	0.100	0.828	0.836	0.830	0.586	0.437	0.499
BM_Ens	0.5	0.113	0.823	0.832	0.825	0.591	0.441	0.502

AUC Area under the receiver operator characteristic curve, ACC predictive accuracy of models at the optimum posterior probability threshold, $R_{nag}^{2}$ Nagelkerke's R^2^, $R_{c\&s}^{2}$ Cox and Snell's R^2^, $R_{ef}^{2}$ Efron's R^2^, RF, Random Forest; SVM, Support Vector Machine; LASSO, Least Absolute Shrinkage and Selection Operator; EN, Elastic Net; ML_Ens, Machine Learning Ensemble; BRR, Bayesian Ridge Regression; BL, Bayesian Lasso; BM_Ens, Bayesian Model ensemble. The number of genome-wide association study risk SNPs considered by each statistical model varied.

### Tuned model performance with and without covariates in the independent validation test set using GWAS top SNPs

Predictive performance with covariates yielded higher ACC for all algorithms after model tuning except RF and results are summarized in Table 2. With covariates and posterior probability threshold tuning, the LASSO and EN algorithms yielded the highest ACC (0.885). Amongst the Bayesian models, the BL and BayesC algorithms yielded the highest ACC (0.842). These models also yielded the highest AUC values. Without covariates, ACC values were lower, and the Bayesian ensemble approach yielded the highest ACC (0.769) and AUC (0.768). Without covariates Bayesian models outperformed machine learning models.

**Table 2:** Polygenic risk score prediction accuracy for cruciate ligament rupture in the Labrador Retriever validation group using Bayesian and machine learning models with and without covariates and top genome-wide risk SNPs.

**Model**	**Optimal threshold after tuning**	P^*^	**gMean**	**ACC**	**AUC**	** $R_{nag\;}^{2}$ **	** $R_{c\&s}^{2}$ **	** $R_{ef}^{2}$ **
**Validation set with covariates**								
RF	0.45	0.048	0.690	0.692	0.690	0.581	0.461	0.492
SVM	0.425	0.024	0.845	0.846	0.845	0.59	0.442	0.511
LASSO	0.5	0.095	0.880	0.885	0.881	0.594	0.444	0.512
EN	0.45	0.018	0.884	0.885	0.884	0.584	0.440	0.502
ML_Ens	0.45	0.018	0.866	0.865	0.866	0.587	0.459	0.506
BRR	0.425	0.125	0.810	0.808	0.813	0.619	0.464	0.525
BL	0.5	0.101	0.841	0.846	0.842	0.616	0.461	0.524
BayesB	0.475	0.065	0.824	0.827	0.824	0.615	0.460	0.523
BayesC	0.475	0.101	0.841	0.846	0.842	0.618	0.462	0.524
BM_Ens	0.5	0.065	0.824	0.827	0.824	0.653	0.489	0.526
**Validation set without covariates**								
RF	0.45	0.030	0.693	0.692	0.693	0.335	0.251	0.243
SVM	0.425	0.107	0.694	0.692	0.696	0.263	0.197	0.194
LASSO	0.5	0.095	0.629	0.635	0.631	0.254	0.190	0.183
EN	0.45	0.018	0.634	0.635	0.634	0.254	0.190	0.183
ML_Ens	0.45	0.048	0.690	0.692	0.690	0.344	0.257	0.250
BRR	0.425	0.001	0.751	0.752	0.751	0.416	0.311	0.347
BL	0.5	0.077	0.746	0.750	0.747	0.41	0.307	0.344
BayesB	0.475	0.077	0.746	0.750	0.747	0.401	0.30	0.335
BayesC	0.475	0.077	0.746	0.750	0.747	0.405	0.303	0.337
BM_Ens	0.5	0.036	0.768	0.769	0.768	0.501	0.374	0.395

AUC Area under the receiver operator characteristic curve, ACC predictive accuracy of models at the optimum posterior probability threshold, $R_{nag\;}^{2}$ Nagelkerke's R^2^, $R_{c\&s}^{2}$ Cox and Snell's R^2^, $R_{ef}^{2}\;$Efron's R^2^, RF, Random Forest; SVM, Support Vector Machine; LASSO, Least Absolute Shrinkage and Selection Operator; EN, Elastic Net; ML_Ens, Machine Learning Ensemble; BRR, Bayesian Ridge Regression; BL, Bayesian Lasso; BM_Ens, Bayesian Model ensemble. The number of genome-wide association study risk SNPs considered by each statistical model varied.

### Predicting cruciate ligament rupture risk using genic SNPs

**Figure 3:**
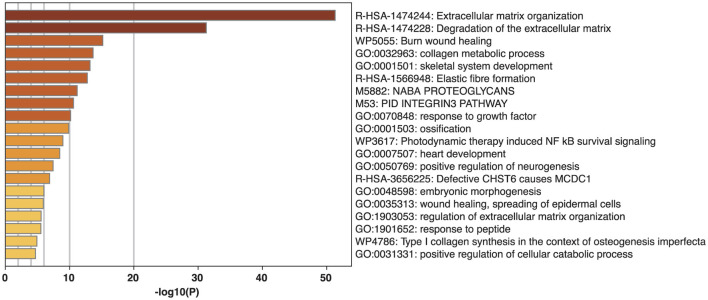
Gene ontology (GO) term and wiki pathway analysis for association with cruciate ligament rupture (CR). The graph compares each GO term's *P*-value for association with CR to the other GO terms. The ID and name for each GO term is listed for each column.

The 41 CR risk genes previously identified in the literature were functionally verified with the GO terms analysis to confirm their relationship with CR. Each GO term represents a particular biological process in the body, and we counted the number of genes from our list that matched each GO term (Figure 3). Amongst the twenty GO terms, extracellular matrix organization (*P* = 4.89E-52), degradation of the extracellular matrix (*P* = 5.5E-32), collagen metabolic process (*P* = 2.3E-14), skeletal system development (*P* = 6.9E-14), elastic fiber formation (*P* = −1.78E-13), response to growth factor (*P* = 6.8E-11), ossification (*P* = 1.38E-10), regulation of the extracellular matrix organization (*P* = 2.75E-6), and Type 1 collagen synthesis (*P* = 1.23E-5) were particularly associated with CR.

PRS prediction was also performed with and without covariates using genic SNPs. With covariates, Bayesian model performance was improved for the BRR, BL, BayesB, and BayesC algorithms, and for the RF and SVM machine learning models, compared with the analysis with genome-wide SNPs (Tables 2, 3). The LASSO and the BL algorithms had the highest ACC (0.875) (Table 3). For the machine learning models with covariates, AUC was highest with the LASSO model (0.871) and for the Bayesian models with covariates the BL model had the highest AUC (0.874). Without covariates, only the LASSO, EN, and ensemble machine learning models exhibited enhanced performance (Tables 2, 3). The LASSO and the EN algorithms had the highest ACC (0.731) without covariates. For the machine learning models without covariates, AUC was highest with the ensemble model (0.731) and for the Bayesian models without covariates the BayesB model had the highest AUC (0.712) (Table 3).

**Table 3:** Polygenic risk score prediction accuracy for cruciate ligament rupture in the Labrador Retriever validation group using Bayesian and machine learning models with and without covariates and genic SNPs.

**Model**	**Optimal threshold after tuning**	P^*^	**gMean**	**ACC**	**AUC**	** $R_{nag\;}^{2}$ **	** $R_{c\&s}^{2}$ **	** $R_{ef}^{2}$ **
**Validation set with covariates using genic SNPs**								
RF	0.45	0.024	0.835	0.836	0.835	0.590	0.442	0.511
SVM	0.425	0.018	0.856	0.855	0.856	0.615	0.460	0.528
LASSO	0.5	0.095	0.870	0.875	0.871	0.629	0.471	0.519
EN	0.45	0.060	0.853	0.855	0.853	0.607	0.454	0.512
ML_Ens	0.45	0.012	0.827	0.827	0.827	0.698	0.522	0.565
BRR	0.425	0.095	0.858	0.855	0.859	0.616	0.461	0.533
BL	0.5	0.018	0.874	0.875	0.874	0.616	0.461	0.534
BayesB	0.475	0.018	0.856	0.855	0.856	0.610	0.457	0.529
BayesC	0.475	0.018	0.856	0.855	0.856	0.614	0.46	0.532
BM_Ens	0.5	0.065	0.824	0.827	0.824	0.635	0.476	0.517
**Validation set without covariates using genic SNPs**								
RF	0.45	0.018	0.634	0.635	0.634	0.244	0.183	0.18
SVM	0.425	0.143	0.675	0.673	0.679	0.249	0.186	0.184
LASSO	0.5	0.119	0.724	0.731	0.726	0.363	0.272	0.278
EN	0.45	0.042	0.729	0.731	0.729	0.317	0.237	0.239
ML_Ens	0.45	0.036	0.732	0.731	0.732	0.437	0.327	0.334
BRR	0.425	0.185	0.693	0.692	0.699	0.290	0.217	0.221
BL	0.5	0.089	0.668	0.673	0.670	0.292	0.218	0.222
BayesB	0.475	0.083	0.707	0.712	0.708	0.281	0.21	0.213
BayesC	0.475	0.030	0.693	0.692	0.693	0.288	0.215	0.219
BM_Ens	0.5	0.244	0.652	0.673	0.664	0.373	0.279	0.273

AUC Area under the receiver operator characteristic curve, ACC predictive accuracy of models at the optimum posterior probability threshold, $R_{nag}^{2}$ Nagelkerke's R^2^, $R_{c\&s}^{2}$ Cox and Snell's R^2^, $R_{ef}^{2}\;$Efron's R^2^, RF, Random Forest; SVM, Support Vector Machine; LASSO, Least Absolute Shrinkage and Selection Operator; EN, Elastic Net; ML_Ens, Machine Learning Ensemble; BRR, Bayesian Ridge Regression; BL, Bayesian Lasso; BM_Ens, Bayesian Model ensemble. The number of genome-wide association study risk SNPs considered by each statistical model varied.

## Discussion

Canine CR is a common orthopaedic disease with a high economic burden from long-term morbidity due to the development of stifle osteoarthritis even in the face of surgical correction (4, 5, 44), so population screening to identify dogs with elevated risk would be an impactful development (5, 45). CR in the Labrador Retriever is a complex heritable disease made up of numerous small effect SNPs and relatively few large effect ones (5). Age of neutering is an important environmental effect (46).

PRS prediction is a powerful tool for defining the heritable risk of developing a disease in an individual subject and is well suited to quantifying a subject's risk for highly complex heritable diseases (19, 20). Such an approach has been extensively used to assess risk of human complex heritable diseases (44). Overall, prediction accuracy was similar between the statistical models we studied. Validation data from the current study suggest PRS prediction of risk of CR in the Labrador Retriever is sufficiently accurate for use as a clinical screening tool for personalized medical care and selection for breeding with a prediction accuracy up to 88% with inclusion of covariates and up to 77% with analysis of only genetic information. Given that PRS prediction only needs a DNA sample easily obtained from a saliva swab, such testing can be performed in puppies before sale to the public, which is potentially advantageous compared with phenotypic screening later in life when dogs may already have been used for breeding or undergone training as a working dog. Additionally, PRS risk prediction testing can provide owners with information that can guide personalized care of the individual dog, particularly regarding modifiable environmental risk factors, such as neutering before 1 year of age (46).

We have recruited a large reference population of Labrador Retrievers over several years that we used as a TRN group for PRS prediction modeling using 10-fold cross validation in the present study. During the initial cross-validation analysis, we found that the optimal SNP set varied amongst the prediction models studied, as previous research has suggested (5). So, our analysis also considered use of an optimal number of SNPs for each model that maximized prediction accuracy. Optimal SNP set size was variable between models with the Bayesian models having the best ability to handle larger number of SNPs in the model training set.

We also found that tuning of the posterior probability threshold led to additional gains in ACC in classifying CR cases from controls, as opposed to using a single threshold of 0.5 for all models (20). In our analysis, we found that ACC and P^*^ did not always align exactly on a specific threshold probability. In this scenario, we emphasized ACC and gMean in our tuning optimization, as ACC is the most clinically relevant parameter describing predictive ability. With the inclusion of individualized optimal posterior probability thresholds, prediction models generally surpassed an ACC of 0.8 with 10-fold cross validation. With our analysis of the validation TST group, model predictions also surpassed an ACC of 0.8 when covariates were included in the model, except for the RF model, suggesting our CR genetic risk prediction approach is a clinically relevant genetic test. Machine learning models, such as RF, require tuning for optimal performance and the weaker performance of this model is likely due to problems with model tuning.

A drawback to 10-fold cross validation, is its tendency to overfit the data, resulting in artificially high PRS scores, because of relatedness between individuals in an inbred population, even if the population is a large one. With our initial 10-fold cross validation within the reference population, ACC was generally above 0.8, but when the validation population was tested without consideration of covariates, ACC fell below 0.7 for machine learning models and below 0.8 for Bayesian models, suggesting overfitting was present in the 10-fold cross validation analysis. This highlights the importance of accounting for population structure and relatedness in predictive modeling, as failure to do so may lead to inflated performance metrics and poor generalizability. Using an external, independent validation group of subjects can help mitigate overfitting by reducing data leakage and ensuring better model robustness. Moreover, consideration of covariates may enhance the validity of PRS prediction, particularly in genetically homogenous or related populations. Covariates are variables that can influence the phenotype independently of genetic risk. Identification of and inclusion of covariates helps separate the contributions of genetic effects from broader physiological or developmental factors and allow the models to more appropriately attribute variation in the results to genetic predictors (47).

Previous work on PRS prediction of CR risk in dogs has shown that the inclusion of covariates in PRS prediction increases accuracy (20). This observation was recapitulated in the present study. We found that the inclusion of sex, neuter status, age, weight, and withers height consistently produced higher predictive accuracy with both our GWAS SNP analysis and the genic only SNP analysis. Without covariates, the highest ACC was 0.769 using a Bayesian ensemble approach. With covariates, the highest ACC was 0.885 using the LASSO and EN machine learning models.

The covariates we considered are readily acquired during routine clinical assessment. This enhances the clinical utility of the PRS models described in this report, as it enables their integration into existing veterinary workflows without the need for additional or specialized clinical assessment or testing. Cost is a significant barrier to veterinary healthcare and obtaining the necessary covariates for our modeling can be done at minimal to no cost to clients. Use of our analytical approach promises early identification of disease risk and provision of timely information for owners by helping to assess a dog's suitability for breeding, or working, and for injury prevention.

We also considered PRS prediction using only genic SNPs. Given that CR is a highly polygenic disease in which risk SNPs are spread throughout the genome (5), we expected limiting the number of SNPs to genic regions would reduce predictive AUC and ACC. We found that the LASSO, EN, and machine learning ensemble models that considered covariates had reduced ACC, but RF and SVM had higher ACC. With the BRR, BL, BayesB, and BayesC models, ACC was also improved by consideration of only genic SNPs, suggesting these models may better capture additive and non-linear effects in genic regions. This could be due to the exclusion of non-genic SNPs reducing statistical noise resulting to enhance signal-to-noise ratio. Genic regions are more likely to contain variants with direct biological relevance making it easier for models with shrinkage or feature selection such as Bayesian or tree-based methods to detect meaningful associations. The finding that performance was reduced with some models when only genic SNPs were considered further supports the notion that the genetic architecture of CR involves both coding and non-coding regulatory elements (48). Collectively our findings suggest both genic and non-genic variants play important, complementary roles in PRS prediction and both need to be considered when conducting PRS analysis (5).

There are several limitations to this research. Our validation population was relatively small compared to the reference population used to train our PRS prediction models. Further expansion of both the TRN and TST groups of dogs would likely further elevate the power of our analysis and provide more robust results. The slight mismatch in the nadir of P^*^ with the peaks in ACC and gMean in our analysis may be indicative of the small sample size used for the validation population. Our analysis only considered the Labrador Retriever. Our gene ontology analysis was based on a candidate gene list that was recently published (17). Other approaches to generation of a gene list could have been used such as genes associated with flanking regions around significant GWAS SNPs. Whilst coat color is known to be associated with CR risk in dogs (18), the coat color phenotype was not available for all dogs in the reference population. Also, GWAS risk SNPs should capture coat color genetic effects. Consequently, coat color was not included as a covariate in our bioinformatics approach to avoid artificially amplifying the risk associated with SNP markers in LD with both coat color and ACL risk.

Previous work from our laboratory suggests there is heterogeneity in the genetic contribution to CR in different breeds of dog (5). Further investigation into this aspect of the genetic contribution to CR is needed in other high-risk breeds such as the Rottweiler and Newfoundland. Ultimately, development of a bioinformatics PRS prediction approach that overcomes this problem would substantially enhance the clinical impact of genetic risk testing for CR in dogs.

In conclusion, our findings suggest that PRS prediction of risk of CR in the Labrador Retriever has sufficient predictive utility for clinical application using only genetic markers with an ACC of 77% with genome-wide SNPs and a Bayesian ensemble approach. We identified further gains in ACC with inclusion of additional readily obtainable clinical covariates yielding an ACC of 88.5% with genome-wide SNPs and a machine learning approach using the LASSO or EN algorithms. Clinically, genetic risk prediction testing has great utility and can be used by breeders during selection for breeding without the need for radiographic testing or waiting years to make an epidemiological determination of the CR status of the dog (23). Additionally, genetic risk testing for CR can be used for screening of individual dogs, particularly working dogs that undertake athletic activity where develop of CR would impair performance. Improved personalized care of the individual patient should focus on correcting modifiable environmental factors in dogs with high genetic risk (46).

## Data Availability

The datasets presented in this study can be found in online repositories. The names of the repository/repositories and accession number(s) can be found below: https://figshare.com/, doi: 10.6084/m9.figshare.28641896 and https://datadryad.org/stash, doi: 10.5061/dryad.47d7wm3ns.
